# Preclinical exploration of combined glucagon inhibition and liver-preferential insulin for treatment of diabetes using in vitro assays and rat and mouse models

**DOI:** 10.1007/s00125-022-05828-w

**Published:** 2022-11-21

**Authors:** Henning Hvid, Christian L. Brand, Tina Hummelshøj, Sanne Jensen, Stephan D. Bouman, Andrew Bowler, Bjarne R. Poulsen, Peter Tiainen, Thorbjörn Åkertröm, Damien Demozay, Thomas Hoeg-Jensen, Camilla Ingvorsen, Thomas Å. Pedersen, Jim McGuire, Thomas Egebjerg, Karen A. Cappelen, Ina P. Eliasen, Bo F. Hansen, Stephanie Hennen, Carsten E. Stidsen, Grith S. Olsen, Nikolaj K. Roed

**Affiliations:** 1 Research & Early Development, Novo Nordisk A/S, Måløv, Denmark; 2 Present Address: QC Laboratories, Syntese A/S, Hvidovre, Denmark; 3 Present Address: Catalyst Biosciences, San Francisco, CA USA; 4 Present Address: Grünethal GmbH, Aachen, Germany

**Keywords:** Alpha cells, Glucagon, Hypoglycaemia, Insulin, Liver, Liver steatosis, Safety

## Abstract

**Aims/hypothesis:**

Normalisation of blood glucose in individuals with diabetes is recommended to reduce development of diabetic complications. However, risk of severe hypoglycaemia with intensive insulin therapy is a major obstacle that prevents many individuals with diabetes from obtaining the recommended reduction in HbA_1c_. Inhibition of glucagon receptor signalling and liver-preferential insulin action have been shown individually to have beneficial effects in preclinical models and individuals with diabetes (i.e. improved glycaemic control), but also have effects that are potential safety risks (i.e. alpha cell hyperplasia in response to glucagon receptor antagonists and increased levels of liver triacylglycerols and plasma alanine aminotransferase activity in response to glucagon receptor antagonists and liver-preferential insulin). We hypothesised that a combination of glucagon inhibition and liver-preferential insulin action in a dual-acting molecule would widen the therapeutic window. By correcting two pathogenic mechanisms (dysregulated glucagon signalling and non-physiological distribution of conventional insulin administered s.c.), we hypothesised that lower doses of each component would be required to obtain sufficient reduction of hyperglycaemia, and that the undesirable effects that have previously been observed for monotreatment with glucagon antagonists and liver-preferential insulin could be avoided.

**Methods:**

A dual-acting glucagon receptor inhibitor and liver-preferential insulin molecule was designed and tested in rodent models (normal rats, rats with streptozotocin-induced hyperglycaemia, *db*/*db* mice and mice with diet-induced obesity and streptozotocin-induced hyperglycaemia), allowing detailed characterisation of the pharmacokinetic and pharmacodynamic properties of the dual-acting molecule and relevant control compounds, as well as exploration of how the dual-acting molecule influenced glucagon-induced recovery and spontaneous recovery from acute hypoglycaemia.

**Results:**

This molecule normalised blood glucose in diabetic models, and was markedly less prone to induce hypoglycaemia than conventional insulin treatment (approximately 4.6-fold less potent under hypoglycaemic conditions than under normoglycaemic conditions). However, compared to treatment with conventional long-acting insulin, this dual-acting molecule also increased triacylglycerol levels in the liver (approximately 60%), plasma alanine aminotransferase levels (approximately twofold) and alpha cell mass (approximately twofold).

**Conclusions/interpretation:**

While the dual-acting glucagon receptor inhibitor and liver-preferential insulin molecule showed markedly improved regulation of blood glucose, effects that are potential safety concerns persisted in the pharmacologically relevant dose range.

**Graphical abstract:**

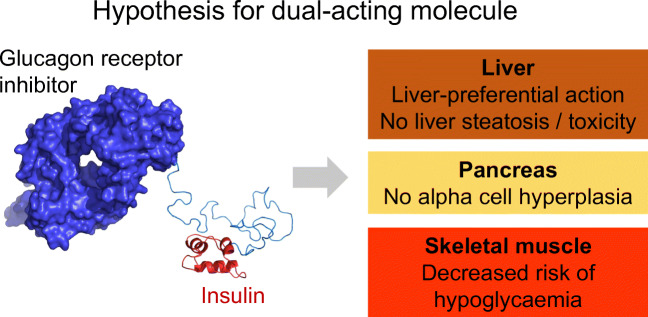

**Supplementary Information:**

The online version contains peer-reviewed but unedited supplementary material available at 10.1007/s00125-022-05828-w.

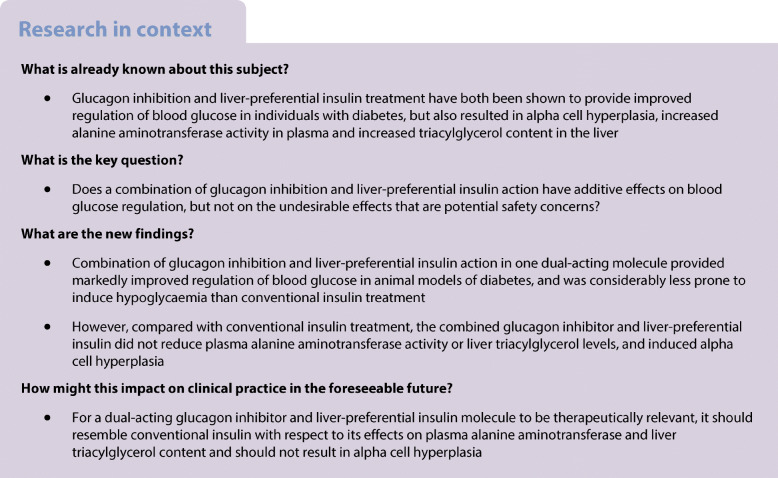



## Introduction

In individuals with diabetes, poor glycaemic control is correlated with increased risk of micro- and macrovascular complications [1]. Several trials (DCCT [2], UKPDS [3], VADT [4], ADVANCE [5] and ACCORD [6]) have consistently demonstrated that intensive glycaemic control with near-normalisation of blood glucose is associated with a lower risk for development of microvascular complications. However, in the ACCORD and ADVANCE trials, intensive glycaemic control did not lead to a reduced risk of development of macrovascular complications, and also increased the incidence of hypoglycaemic events [5, 6]. Whether hypoglycaemic events and macrovascular complications are directly linked is a matter of some debate [7–9]. However, intensive glycaemic control with a target HbA_1c_ <48 mmol/mol (6.5%) is in any case not recommended as the standard of care for all patients [10], and diabetes treatment that normalises blood glucose without causing hypoglycaemia is expected to reduce risk of complications.

Type 1 and type 2 diabetes are both characterised by impaired glucagon secretion, which fails to counteract hypoglycaemia and contributes to hyperglycaemia [11–15]. Furthermore, when individuals with diabetes are treated with insulin by s.c. injection, lower levels of insulin reach the liver and higher levels reach peripheral tissues compared with the distribution of endogenous insulin in healthy individuals [16]. Thus, the combination of impaired secretion of glucagon and non-physiological distribution of injectable insulin contributes to a higher risk of hypoglycaemic events and long-term micro- and macrovascular complications in diabetes patients [16, 17]. Correction of these issues would improve both fasting and prandial glucose regulation and reduce the risk of hypoglycaemia.

Glucagon receptor (GCGR) antagonists have been shown to improve glucose regulation without increased risk of hypoglycaemia in both type 1 and type 2 diabetes [18–20], and to increase plasma levels of glucagon-like peptide 1 and glucagon, possibly as a result of alpha cell hyperplasia, which results in increased glucagon-like peptide 1/glucagon production/processing in pancreatic alpha cells [18, 21]. GCGR antagonists have also been associated with increased triacylglycerol (TAG) levels in the liver and elevated plasma alanine aminotransferase (ALT) activity, suggesting liver damage [18, 19, 21]. Thus, GCGR antagonists have both beneficial and undesirable effects in patients. Beneficial and unwanted effects were also observed for the liver-preferential basal insulin lispro (BIL), which was reported to reduce the risk of hypoglycaemia [22] but at the same time was associated with increased plasma ALT activity and elevated levels of liver TAG in insulin-naive patients, which are known safety concerns for liver-preferential insulin [23].

These findings led us to hypothesise that treatment with a molecule that combined a GCGR antagonist and liver-preferential insulin would widen the therapeutic window, i.e. have the same beneficial effects as observed when the treatment regimens were used alone, and at the same time have diminished unwanted effects. As explained above, dysregulated glucagon signalling and non-physiological insulin treatment both contribute to impaired glycaemic control. A novel treatment regimen with combined correction of these two pathogenic mechanisms may be hypothesised to require lower doses of each component (i.e. GCGR antagonist and liver-preferential insulin) for normalisation of blood glucose compared to monotreatment with either a GCGR antagonist or liver-preferential insulin, and thereby not result in the unwanted effects of alpha cell hyperplasia, increased liver fat and increased plasma ALT activity observed previously for GCGR antagonists and liver-preferential insulin. In fact, it has been reported that synergistic combination of compounds in general can overcome toxic or other unwanted adverse effects [24]. The aim of the present study was therefore to engineer a dual-acting molecule with combined GCGR antagonistic effect and liver-preferential insulin action and explore its pharmacological effects. The liver-preferential effect of insulin would result from the liver-preferential expression of GCGR. The present study describes the structure and pharmacological properties of this dual-acting molecule, with special focus on potential safety concerns.

## Methods

In addition to the descriptions below, relevant detailed descriptions of materials, methods, assays and animal experiments are available in the electronic supplementary material (ESM Methods).

### In vitro experiments

#### Compounds

**Fig. 1:**
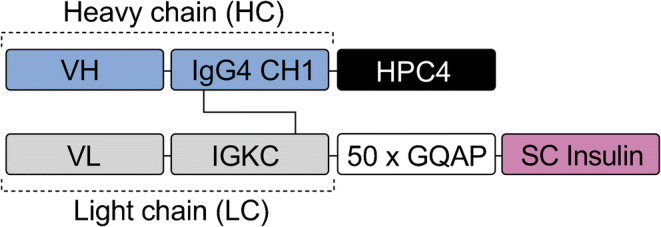
Composition of the GCGRi–insulin molecule. The molecule comprised a human inhibitory anti-GCGR Fab region including heavy chain (HC) and light chain (LC) variable regions from the anti-GCGR Amgen A3 antibody [25]. The Fab fragment was fused to residue B1 of a single-chain insulin variant precursor (consisting of a B-chain [B1–B29] linked to an A-chain [A1–A21] by a connecting peptide [TGLGSGK]) via a 200 amino acid linker (50 GQAP sequence repeats) between the C-terminus of the Fab light chain and insulin B1. SC, single chain; VH, heavy chain variable domain; VL, light chain variable domain; CH1, heavy chain constant domain 1; IGKC, immunoglobulin kappa constant domain; HPC4, protein C epitope tag peptide

The GCGR inhibitor (GCGRi)–insulin fusion molecule comprised a human inhibitory anti-GCGR fragment antigen-binding (Fab) region including heavy chain and light chain variable regions from the anti-GCGR Amgen A3 antibody (referred to as A-3 in US patent US-7947809-B2 [25]). The Fab fragment was fused to residue B1 of a single-chain insulin variant precursor (consisting of a B-chain [B1–B29] linked to an A-chain [A1–A21] by a connecting peptide [TGLGSGK]) via a 200 amino acid linker (50 GQAP sequence repeats) [26] between the C-terminus of the Fab light chain and insulin B1 (Fig. 1). The sequence of the Fab fragment was chosen based on its high affinity and high inhibitory potency, to ensure targeting of the compound to the liver and inhibition of GCGR (Table 1). A fusion molecule was created for use as a negative control (NC), comprising the single-chain insulin precursor variant used in the GCGRi–insulin molecule and an anti-trinitrophenyl Fab fragment instead of the anti-GCGR Fab fragment described above for the GCGRi–insulin molecule. The anti-trinitrophenyl Fab fragment and single-chain insulin precursor in the NC were fused via a 200 amino acid linker with 50 GQAP repeats.

**Table 1:** Overview of the animal experiments

Experiment	Aims	Results described in
A	To compare the effect of a single treatment (i.v. injection) with GCGRi–insulin, NC or GCGRi on blood glucose in STZ-treated rats	Fig. 2a–c
B	To compare the effect of a single treatment (i.v. injection) with GCGRi–insulin with the effect of conventional insulin treatment (I700) on blood glucose in *db*/*db* mice	Fig. 2d–f
C	To compare the response to glucagon (i.v. bolus) between rats in which hypoglycaemia was induced by constant i.v. infusion with either GCGRi–insulin or conventional insulin (HI)	Fig. 3a–c, Table 3
D	To compare the spontaneous recovery from hypoglycaemia induced by an i.v. bolus of fast-acting insulin in rats in which plasma glucose had been decreased to 4 mmol/l (normoglycaemia) by constant i.v. infusion of either GCGRi–insulin or conventional insulin (HI)	Fig. 3d–g, Table 3
E	To compare the doses (i.e. infusion rates) required to decrease plasma glucose in rats to 3 or 4 mmol/l by constant i.v. infusion of either conventional insulin (HI or I700) or liver-preferential insulin (BIL)	Table 3
F	To compare the effect of treatment (s.c. injection) for 21 days with either GCGRi–insulin or conventional insulin (I501) on glucose uptake (i.e. 2-deoxy-glucose) in adipose tissue and skeletal muscle, plasma NEFA, plasma ALT, liver TAG content and alpha cell mass, at matched blood glucose-lowering doses in STZ-DIO mice	Figs 4 and 5

Native human insulin (HI) and the long-acting insulin analogues insulin 501 (I501, desB30 HI conjugated at B29Lys with octadecandioic acid via a γ-glutamic acid linker), insulin 700 (I700, HI conjugated at B29Lys with hexadecandioic acid via a γ-glutamic acid linker) [27] and BIL [28] used as comparators in this study were produced by Novo Nordisk (Denmark). I700 was used as a comparator because it has pharmacodynamic and pharmacokinetic characteristics that are fully comparable to those of insulin degludec (data not shown), a well-characterised insulin analogue that is used in clinical practice, which it was important to compare GCGRi–insulin with. I501 was used as a comparator because, upon s.c. injection in mice, it had pharmacokinetic characteristics comparable to those of s.c. injected GCGRi–insulin (data not shown). Furthermore, I501 was used in a dose that had a similar effect on blood glucose as the chosen dose of GCGRi–insulin. It was important to ensure that GCGRi–insulin and the conventional insulin comparator had comparable pharmacokinetic/pharmacodynamic effects when comparing the effects of the two compounds on liver TAG content and plasma ALT activity, as differences in pharmacokinetic effect and effects on blood glucose regulation in themselves influence liver TAG and ALT activity. The HI, I700, I501, BIL and glucagon (Hypokit, Novo Nordisk) used in the animal experiments were formulated in 5 mmol/l phosphate, 140 mmol/l NaCl, 70 ppm polysorbate 20, pH 7.4. GCGRi–insulin, GCGRi and the NC (Novo Nordisk) were formulated in 25 mmol/l histidine and 150 mmol/l NaCl.

#### Insulin receptor and glucagon receptor binding

Affinities for human insulin receptor isoforms A and B (hIR-A and hIR-B) were determined by scintillation proximity radio-ligand competition binding assays as previously reported [29]. Competitive ^125^I-glucagon binding was performed on plasma membranes prepared from baby hamster kidney (BHK) cells expressing human GCGR and Cre recombinase–firefly luciferase.

#### Inhibition of glucagon receptor signalling in BHK cells

Inhibition of GCGR signalling was determined in the presence of 20 pmol/l glucagon (Novo Nordisk) in BHK cells stably overexpressing human GCGR and cAMP-sensitive Cre recombinase–firefly luciferase (Novo Nordisk).

#### Insulin receptor signalling in primary rat hepatocytes

Hepatocytes from ad libitum-fed male Sprague Dawley rats (body weight 200 g) were isolated in situ by a two-step perfusion technique as described previously [30]. The following day, the primary hepatocytes were used for assessment of insulin receptor (IR) phosphorylation (p-Tyr1150/1151) or Akt phosphorylation (p-Ser473) upon stimulation with increasing concentrations of HI, the dual GCGRi–insulin compound or the NC for 15 min, using the AlphaScreen SureFire assay kit (Perkin-Elmer, Denmark).

#### Inhibition of glucagon receptor signalling in primary rat hepatocytes

Primary rat hepatocytes were incubated with increasing concentrations of the dual GCGRi–insulin compound or GCGRi in the presence of 3 nmol/l glucagon for 30 min, and cAMP production in the cells was subsequentially quantified using a FlashPlate assay kit (Perkin-Elmer).

#### Glycogen synthesis in primary hepatocytes

Primary rat hepatocytes were incubated in Medium 199 (Gibco, Denmark, see ESM Methods for detailed description) containing 14.5 mmol/l glucose for 24 h with increasing concentrations of HI, the dual GCGRi–insulin compound or the NC. Cells were then lysed by freezing in liquid nitrogen and treated with amyloglucosidase, and cellular glucose content (glycogen) was quantified using a BioVision glucose assay kit (BioVision Research Products, USA).

#### Lipogenesis in primary mouse adipocytes

The effect of GCGRi–insulin and NC on lipogenesis was determined in primary mouse adipocytes isolated from epididymal fat pads by measuring the incorporation of [3H]-labelled glucose into fat as described previously [31].

### Animal experiments

This study comprised six animal experiments (experiments A–F; see Table 1 and ESM Table 1). All animals included in this study were male and housed in type IV Makrolon cages with unrestricted access to a pelleted rodent diet (Altromin 1324 [Brogården, Denmark] in experiments A–E; high-fat diet D12492 [with 60% energy from fat; Research Diets, USA] in experiment F) and tap water (non-chlorinated, non-acidified). The animals were housed at 18–24°C, with relative humidity 30–70%, air change 8–15 times/h and a light/dark cycle of 12 h/12 h. All animals were acclimatised for at least a week prior to initiation of experimental procedures, which were ethically approved by the Danish Animal Experiments Inspectorate. In brief, acute effects on blood glucose of a single treatment with a bolus of GCGRi–insulin, NC, GCGRi or the basal insulin comparator I700 were explored in Sprague Dawley rats (10–11 weeks old) made hyperglycaemic using streptozotocin (STZ) and in *db*/*db* mice (BKS.Cg-Dock7m+/+*Lepr*^*db*^J; strain number 000642; The Jackson Laboratory, USA) (10–11 weeks old). Sprague Dawley rats (12–13 weeks old) were also used in experiments in which GCGRi–insulin, HI, I700 or BIL were infused i.v. to a target plasma glucose of 3 or 4 mmol/l (i.e. hypoglycaemia). After reaching the target plasma glucose, rats were challenged using either a bolus of vehicle, a bolus of glucagon or a bolus of fast-acting HI, to assess recovery from hypoglycaemia. The infusion rates of HI, I700, BIL or GCGRi–insulin from 90–180 min in rats given vehicle at 90 min in the 3 and 4 mmol/l glucose studies were used for a post hoc analysis. The effects of the basal insulin comparator I501 or GCGRi–insulin on blood glucose, 2-deoxy-glucose uptake in adipose tissue and skeletal muscle, HbA_1c_, plasma NEFA levels, plasma ALT activity, liver TAG levels and pancreatic alpha cell mass after treatment for 3 weeks were explored in C57BL/6J mice (strain number 380050; The Jackson Laboratory, USA) with diet-induced obesity and STZ-induced hyperglycaemia (STZ-DIO mice). The STZ-DIO mice were 16 weeks old when treatment was started.

#### Histology

The fixed pancreas was weighed, processed for paraffin embedding, and randomly cut into four slabs for stereological assessment of alpha and beta cell mass. Alpha cells were detected using a mouse anti-glucagon antibody (Novo Nordisk), and beta cells were visualised using a guinea pig anti-insulin antibody (Dako, Denmark). The stained slides were scanned using a VS120 slide scanner (Olympus, Germany). Total tissue area, glucagon area (alpha cells) and insulin area (beta cells) were quantified in the digital images using VIS software (Visiopharm, Denmark). Alpha and beta cell mass were estimated by multiplying the fractional areas by the total pancreas mass as described previously [32].

### Statistical analysis

Statistical analysis was performed using GraphPad Prism (GraphPad Software, USA), JMP (SAS Institute, USA) or SAS software (SAS Institute). All in vitro data were fitted to a four-parameter logistic model, and the estimated EC_50_/IC_50_ values were subsequently used for calculation of geometric mean values and 95% CI. In vivo data were analysed using general linear models, followed by pairwise comparison of treatment groups, with Tukey’s adjustment for multiple parallel pairwise comparisons. Data were log-transformed prior to analysis if assumptions of normal distribution and/or variance homogeneity were not fulfilled. The individual molar infusion rates required to obtain plasma glucose of 3 or 4 mmol/l in experiments C, D and E were used in a post hoc analysis. All data were log-transformed and analysed using two-way ANOVA with interaction between the factors compound and plasma glucose target level. Separate variances were estimated to take variance inhomogeneity into account. Furthermore, differences between each compound and HI were estimated on the log scale. The double difference (i.e. [compound X vs HI at 3 mmol/l] vs [compound X vs HI at 4 mmol/l]) was also estimated on the log scale. Estimated differences and double differences on the log scale were back-transformed to ratios (i.e. [compound X at 3 mmol/l/HI at 3 mmol/l]/[compound X at 4 mmol/l/HI at 4 mmol/l]). In all analyses, *p* values <0.05 were considered statistically significant.

## Results

### The GCGRi–insulin compound bound to and inhibited the GCGR

Fusion of insulin to the GCGRi Fab fragment did not influence the binding affinity of GCGRi to the GCGR (Table 2). The inhibitory potency of GCGRi–insulin was found to be only slightly lower than that of GCGRi in BHK cells expressing human GCGR as well as in primary rat hepatocytes (Table 2).

**Table 2:** Receptor binding and activation characteristics of the GCGRi–insulin molecule

Compound	hGCGR binding (IC_50_)	hGCGR inhibition in BHK cells (20 pmol/l glucagon) (IC_50_)	GCGR inhibition in rat hepatocytes (3 nmol/l glucagon) (IC_50_)	hIR-A binding (IC_50_)	hIR-B binding (IC_50_)	IR phosphorylation (EC_50_)	Akt phosphorylation (EC_50_)	Glycogen synthesis in rat hepatocytes (EC_50_)	Lipogenesis in mouse adipocytes (EC_50_)
Glucagon	0.79 (0.67, 0.93)	–	–	–	–	–	–	–	–
GCGRi	0.46 (0.32, 0.66)	1.55 (0.84, 2.86)	16.5 (10.2, 26.7)	–	–	–	–	–	–
HI	–	–	–	0.51 (0.44, 0.59)	0.73 (0.59, 0.90)	0.82 (0.24, 2.82)	1.05 (0.36, 3.03)	0.47 (0.26, 0.84)	0.05 (0.02, 0.11)
HI + 10 μmol/l GCGRi	–	–	–	–	–	0.98 (0.23, 4.23)	0.63 (0.17, 2.38)	0.59 (0.11, 3.22)	–
GCGRi–insulin	0.40 (0.27, 0.61)	4.7 (4.0, 5.7)	55.9 (41.0, 76.2)	963.7 (796.1, 1167.0)	2022.0 (1369.0, 2987.0)	53.5 (6.9, 414.8)	31.7 (12.7, 78.7)	3.72 (2.75, 5.03)	126.2 (65.7, 242.2)
GCGRi–insulin + 10 μmol/l GCGRi	–	–	–	–	–	1040.0 (322.1, 3357.0)	641.7 (461.2, 892.8)	78.0 (23.8, 255.6)	–
NC	–	–	–	907.9 (711.3, 1159.0)	1386.0 (689.1, 2788.0)	5019 (1390, 18,121)	763.7 (407.1, 1433.0)	199.2 (94.9, 418.0)	152.3 (115.3, 201.0)
NC + 10 μmol/l GCGRi	–	–	–	–	–	5127 (4320, 6084)	1249.0 (761.2, 2049.0)	140.9 (47.6, 417.2)	–

Data are geometric means and 95% CI. All values are nmol/l

hGCGR, human GCGR

### GCGRi–insulin and the NC bound to the IR with similar affinity in the absence of GCGR

The binding affinities of GCGRi–insulin and the corresponding NC for hIR-A and hIR-B were fully comparable at approximately 0.05% relative to HI for hIR-A [(1/[IC_50_(GCGRi–insulin)/IC_50_(HI)]) × 100%; see Table 2] and approximately 0.04% relative to HI for hIR-B [(1/[IC_50_(GCGRi–insulin)/IC_50_(HI)]) × 100%; see Table 2].

### GCGRi–insulin induced signalling and glucose metabolic effects in hepatocytes with higher potency than the negative control

GCGRi–insulin induced a concentration-dependent increase in IR phosphorylation with much higher potency (EC_50_ 54 nmol/l) than NC (EC_50_ 5019 nmol/l) (Table 2). In the presence of 10 μmol/l GCGRi, the potency of GCGRi–insulin decreased towards the potency of NC (Table 2). By contrast, the concentration–response curves for NC remained unchanged in the presence of the antagonist, as this NC only binds to the IR. This indicated that binding of the dual GCGRi–insulin compound to both the GCGR and IR was necessary to achieve the higher IR potency in hepatocytes, as described previously for a fusion molecule of insulin and an anti-asialoglycoprotein receptor antibody [33]. Concentration–response curves also revealed that GCGRi–insulin had a higher potency with respect to activation of Akt, found downstream of the IR (EC_50_ 32 nmol/l), compared with NC (EC_50_ 764 nmol/l) (Table 2). GCGRi–insulin also stimulated glycogen synthesis in hepatocytes with a higher potency than NC (EC_50_ 3.72 nmol/l and 199.2 nmol/l, respectively) (Table 2). In primary mouse adipocytes, which do not express the GCGR, GCGRi–insulin and NC stimulated lipogenesis with similar potency (approximately 0.04% of that of HI) (Table 2). Taken together, the in vitro data demonstrate that the GCGRi–insulin molecule had much higher potency than the NC in hepatocytes (which express GCGR) compared with adipocytes (which do not express GCGR). This finding supported the hypothesis that GCGRi–insulin molecule would have a liver-preferential effect in vivo.

### A single injection of GCGRi–insulin induced prolonged blood glucose lowering in hyperglycaemic rats and mice

**Fig. 2:**
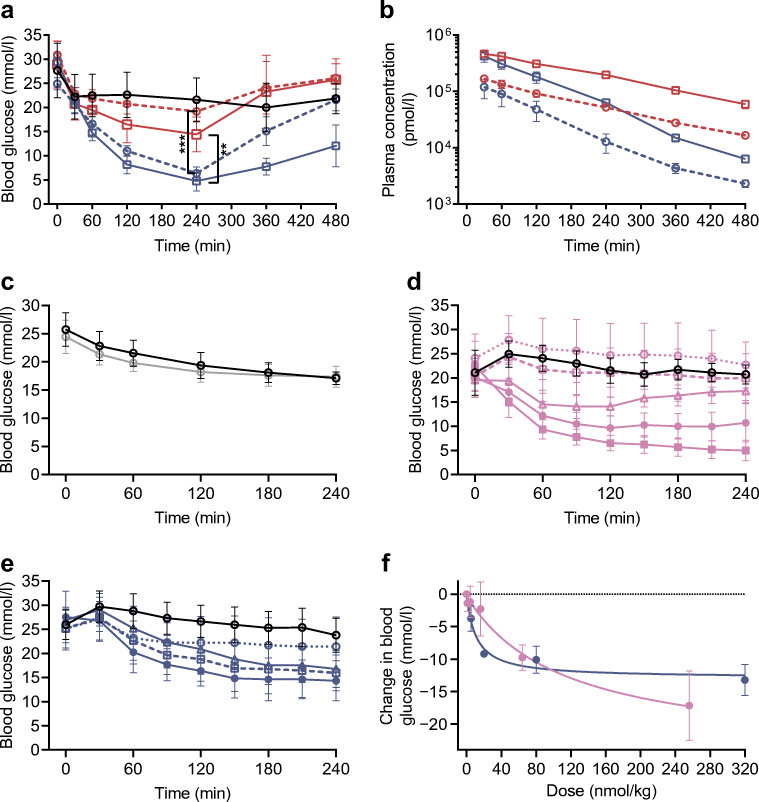
Effect on blood glucose in hyperglycaemic STZ-treated rats and *db*/*db* mice. (**a**) Blood glucose in hyperglycaemic STZ-treated rats after treatment by i.v. injection with vehicle (black circles, *n*=5), 10 nmol/kg GCGRi–insulin (blue circles, *n*=5), 30 nmol/kg GCGRi–insulin (blue squares, *n*=5), 10 nmol/kg NC (red circles, *n*=5) or 30 nmol/kg NC (red squares, *n*=5). Values are means ± SD. **p<0.01, ***p<0.001 as indicated. (**b**) Plasma concentrations of test compounds measured in hyperglycaemic STZ-treated rats after treatment by i.v. injection with 10 nmol/kg GCGRi–insulin (blue circles, *n*=5), 30 nmol/kg GCGRi–insulin (blue squares, *n*=5), 10 nmol/kg NC (red circles, *n*=5) or 30 nmol/kg NC (red squares, *n*=5). Values are means ± SD, *y*-axis is logarithmic (log_10_). (**c**) Blood glucose in hyperglycaemic STZ-treated rats after treatment by i.v. injection with vehicle (black circles, *n*=5) or 60 nmol/kg GCGRi (grey circles, *n*=5). Values are means ± SD. (**d**) Blood glucose in hyperglycaemic *db*/*db* mice after treatment by i.v. injection with vehicle (black circles, *n*=8) or 1 nmol/kg (open pink circles, *n*=8), 4 nmol/kg (open pink squares, *n*=8), 16 nmol/kg (pink triangles, *n*=8), 64 nmol/kg (closed pink circles, *n*=8) or 256 nmol/kg (closed pink squares, *n*=10) of I700. Values are means ± SD. (**e**) Blood glucose in hyperglycaemic *db*/*db* mice after treatment by i.v. injection with vehicle (black circles, *n*=8) or 5 nmol/kg (open blue circles, *n*=5), 20 nmol/kg (blue squares, *n*=5), 80 nmol/kg (blue triangles, *n*=5) or 320 nmol/kg (closed blue circles, *n*=5) of GCGRi–insulin. Values are means ± SD. (**f**) Change in blood glucose 240 min after treatment, plotted against the i.v. administered dose of I700 (closed pink circles: 1 nmol/kg, *n*=8; 4 nmol/kg, *n*=8; 16 nmol/kg, *n*=8; 64 nmol/kg, *n*=8; 256 nmol/kg, *n*=10) or GCGRi–insulin (closed blue circles: 5 nmol/kg, *n*=5; 20 nmol/kg, *n*=5; 80 nmol/kg, *n*=5; 320 nmol/kg, *n*=5). The lines indicate the dose–response relationship for I700 (pink) and GCGRi–insulin (blue)

GCGRi–insulin decreased blood glucose 0–240 min after treatment in a dose-dependent manner and significantly more than equimolar doses of the NC in hyperglycaemic STZ-treated rats 240 min after treatment (*p*=0.005 and *p*<0.0001, respectively; Fig. 2a). As the plasma concentration of the NC was comparable or higher than that of GCGRi–insulin (Fig. 2b), the GCGRi–insulin appeared more potent than the NC in vivo. This was probably caused by a liver-preferential effect of the GCGRi–insulin, as treatment with vehicle or GCGRi alone at 60 nmol/kg did not decrease blood glucose in STZ-treated rats (Fig. 2c). It is perhaps surprising that treatment with 60 nmol/kg GCGRi did not decrease blood glucose in STZ-treated rats. This is probably due to low levels of glucagon, which in this model does not have a major impact on hepatic glucose output. In other animal models, the GCGRi had a minor effect on blood glucose, comparable to that of the NC (data not shown).

GCGRi–insulin also decreased blood glucose in a dose-dependent manner in *db*/*db* mice (Fig. 2e). Interestingly, GCGRi–insulin decreased blood glucose more potently than conventional basal I700 at low to medium doses, but the effect on blood glucose was less potent than that of I700 at doses above 20 nmol/kg (Fig. 2d–f). This observation supports the hypothesis that the GCGRi–insulin molecule had a liver-preferential action, as lower potency of the compound at higher doses may be a result of lower IR activation in peripheral tissues such as skeletal muscle and adipose tissue. Furthermore, this observation indicates that GCGRi–insulin may be less prone to induce hypoglycaemia than traditional insulin treatment.

### Recovery from hypoglycaemia using glucagon occurred more rapidly when hypoglycaemia was induced by GCGRi–insulin compared with HI

**Fig. 3:**
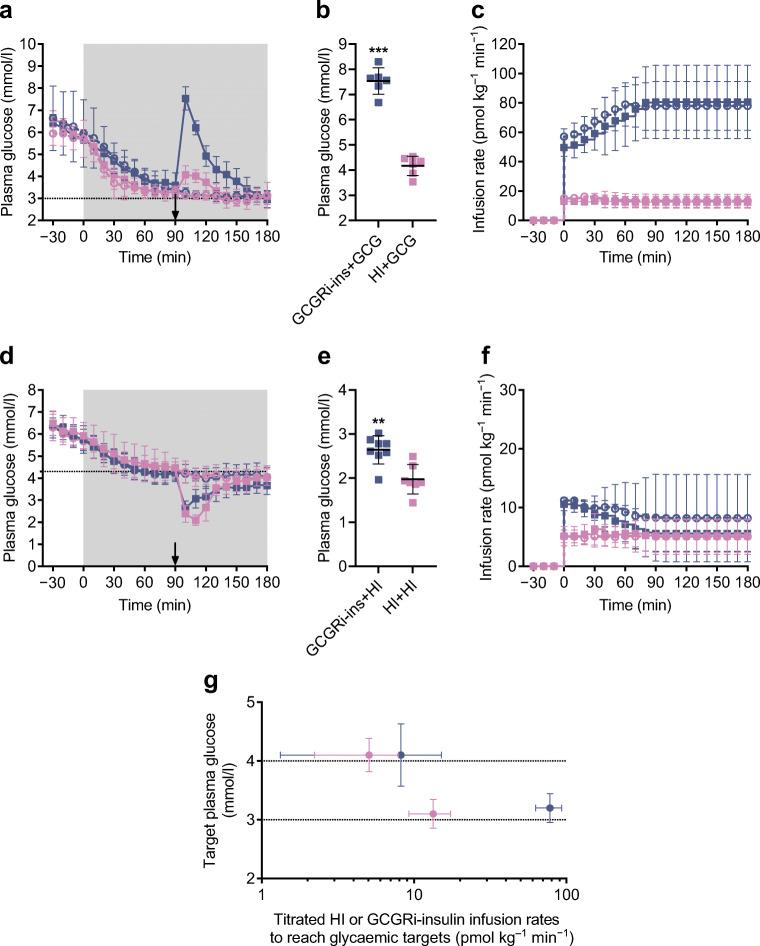
Recovery from acute hypoglycaemia. (**a**) Reduction of plasma glucose to a target of 3 mmol/l by i.v. infusion of HI (pink symbols) or GCGRi–insulin (blue symbols). After 90 min (arrow), the rats treated with HI by constant i.v. infusion received an i.v. bolus of 10 nmol/kg glucagon (closed pink squares, *n*=6) or vehicle (open pink circles, *n*=6). The rats treated with GCGRi–insulin by constant i.v. infusion also received an i.v. bolus of 10 nmol/kg glucagon (closed blue squares, *n*=6) or vehicle (open blue circles, *n*=6) at 90 min. Values are means ± SD. The grey-shaded area indicates the period with i.v. infusion of HI or GCGRi–insulin. (**b**) Plasma glucose levels 10 min after administration of a glucagon bolus to rats receiving GCGRi–insulin (blue squares, *n*=6) or HI (pink squares, *n*=6) by i.v. infusion (i.e. 100 min after the start of infusion). Symbols indicate observations from individual animals. Horizontal lines indicate means; bars indicate SD. ***p<0.001 vs the HI-treated group. (**c**) Infusion rates in rats administered either HI and an i.v. bolus of vehicle (open pink circles, *n*=6), HI and an i.v. bolus of glucagon (closed pink squares, *n*=6), GCGRi–insulin and an i.v. bolus of vehicle (open blue circles, *n*=6) or GCGRi–insulin and an i.v. bolus of glucagon (closed blue squares, *n*=6). Values are means ± SD. (**d**) Reduction of plasma glucose to a target of 4 mmol/l by i.v. infusion of HI (pink symbols) or GCGRi–insulin (blue symbols). After 90 min (arrow), the rats treated with HI by constant i.v. infusion received an i.v. bolus of 1.1 nmol/kg HI (closed pink squares, *n*=7) or vehicle (open pink circles, *n*=8). The rats treated with GCGRi–insulin by constant i.v. infusion also received an i.v. bolus of 1.1 nmol/kg HI (closed blue squares, *n*=8) or vehicle (open blue circles, *n*=7) at 90 min. Values are means ± SD. The grey-shaded area indicates the period with i.v. infusion of HI or GCGRi–insulin. (**e**) Plasma glucose levels 20 min after administration of an i.v. bolus of HI to rats receiving GCGRi–insulin (blue squares, *n*=8) or HI (pink squares, *n*=7) by constant i.v. infusion (i.e. 110 min after the start of infusion). Symbols indicate observations from individual animals. Horizontal lines indicate means; bars indicate SD. **p<0.01 vs the HI-treated group. (**f**) Infusion rates in rats administered either HI and an i.v. bolus of vehicle (open pink circles, *n*=8), HI and an i.v. bolus of HI (closed pink squares, *n*=7), GCGRi–insulin and an i.v. bolus of vehicle (open blue circles, *n*=7) or GCGRi–insulin and an i.v. bolus of HI (closed blue squares, *n*=8). Values are means ± SD. (**g**) Plasma glucose plotted against infusion rate for rats treated with HI (pink symbols: plasma glucose 3 mmol/l, *n*=6; plasma glucose 4 mmol/l, *n*=8) or GCGRi–insulin (blue symbols: plasma glucose 3 mmol/l, *n*=6; plasma glucose 4 mmol/l, *n*=7). Values are means ± SD

Glucagon-mediated recovery from hypoglycaemia induced by constant infusion with either HI or GCGRi–insulin was explored in rats (Fig. 3a–c). Surprisingly, plasma glucose increased significantly more upon glucagon challenge in the group of rats in which hypoglycaemia was induced using GCGRi–insulin (*p*<0.001), and plasma glucose was elevated for a longer time compared with animals in which hypoglycaemia was induced using HI (Fig. 3a,b). A possible explanation may be that GCGRi–insulin inhibited the action of endogenous glucagon. When challenged with exogenous glucagon, a larger amount of glycogen would consequently be available for glycogenolysis, compared with the group in which hypoglycaemia was induced using HI.

### GCGRi–insulin was less prone to cause hypoglycaemia following a bolus of HI

The ability of endogenous counter-regulatory mechanisms to rescue acute hypoglycaemia induced by fast-acting insulin was explored in rats in which blood glucose was reduced to 4 mmol/l by constant infusion of either GCGRi–insulin or HI, and a bolus of HI was subsequently administered (Fig. 3d–f). This set-up thereby mimicked accidental overdose with fast-acting insulin in an individual who is also using basal insulin treatment. The bolus of fast-acting HI decreased plasma glucose significantly less in the rats receiving a constant infusion of GCGRi–insulin compared with the group given HI by constant infusion (*p*=0.0017, Fig. 3e).

### Constant infusion of GCGRi–insulin induced hypoglycaemia less potently than HI

The molar infusion rates of GCGRi–insulin required to decrease plasma glucose to 3 and 4 mmol/l were compared with the corresponding infusion rates of HI (Fig. 3g and Table 3). To obtain a plasma glucose of 4 mmol/l, the required infusion rate of GCGRi–insulin was 1.3-fold higher than for HI. However, the infusion rate of GCGRi–insulin required to reach the hypoglycaemic target of 3 mmol/l was 6.0-fold higher than for HI, i.e. to obtain a hypoglycaemic plasma glucose of 3 mmol/l, the dose of GCGRi–insulin had to be increased approximately 4.6-fold more than required to obtain a plasma glucose of 4 mmol/l. This double ratio of 4.6 was significantly different from 1.0 (*p*=0.0025, Table 3), and demonstrated that GCGRi–insulin was significantly less potent relative to HI under hypoglycaemic conditions (relative potency of approximately 17%) than under normoglycaemic conditions (relative potency of approximately 77%). When tested in the same experimental set-up, there was, as expected, no significant difference in the doses of I700 required to obtain plasma glucose levels of 3 and 4 mmol/l compared with HI (Table 3). For BIL, calculation of the double ratio showed that the dose had to be increased 2.4-fold more to reach 3 mmol/l than to reach 4 mmol/l compared with HI (Table 3). This non-significant difference (*p*=0.0766) is in good agreement with previous studies showing that BIL was liver-preferential and caused fewer hypoglycaemic events than conventional insulin treatment in individuals with diabetes [22, 34, 35].

**Table 3:** Absolute and relative infusion rates to reach normoglycaemic and hypoglycaemic plasma glucose for GCGRi–insulin and comparators

Compound	Infusion rate to reach target plasma glucose (pmol kg–1 min−1)		Infusion rate relative to HI (ratio)		Double ratio with 95% CI^a^	*p*
	3 mmol/l	4 mmol/l	3 mmol/l	4 mmol/l		
HI	12.7 (9.0, 18.0)^b^ 10.4 (6.3, 17.0)^c^	4.5 (2.9, 6.9)^b^ 5.0 (2.7, 9.3)^c^	–	–	–	–
I700	31.0 (20.2, 47.8)^c^	16.9 (9.1, 31.3)^c^	3.0 (1.7, 5.2)^c^	3.4 (1.7, 6.5)^c^	0.9 (0.4, 2.1)^c^	0.7933
BIL	79.0 (45.4, 137.3)^c^	16.2 (8.6, 30.8)^c^	7.6 (3.8, 15.3)^c^	3.2 (1.7, 6.2)^c^	2.4 (0.9, 6.1)^c^	0.0766
GCGRi–insulin	76.5 (61.3, 95.4)^b^	5.9 (2.6, 13.0)^b^	6.0 (4.2, 8.6)^b^	1.3 (0.6, 3.0)^b^	4.6 (1.9, 11.0)^b^	0.0025

Data for infusion rate are geometric means and 95% CI

^a^The double ratio was calculated as [compound X at 3 mmol/l/HI at 3 mmol/l]/[compound X at 4 mmol/l/HI at 4 mmol/l]

^b^Data from experiments C and D

^c^Data from experiment E

### GCGRi–insulin had a diminished effect on glucose uptake in adipose tissue and skeletal muscle

**Fig. 4:**
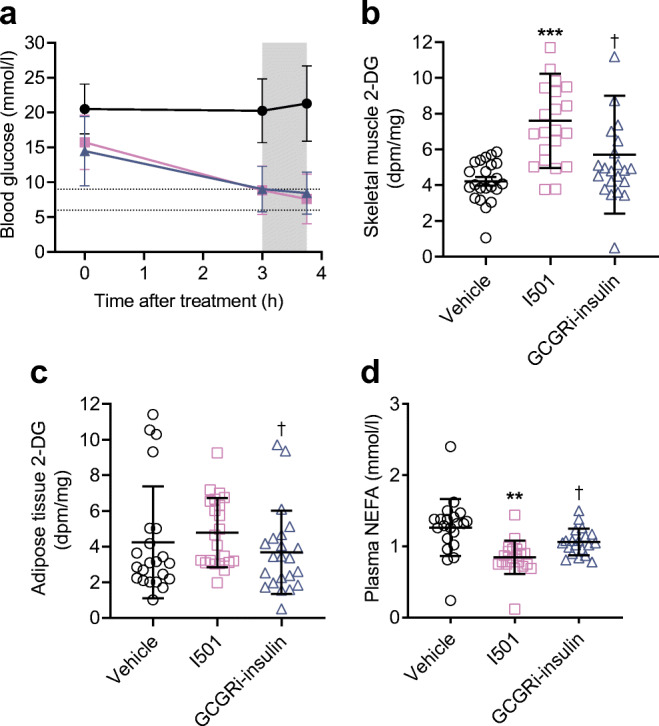
Blood glucose levels, 2-deoxy-glucose (2-DG) uptake in skeletal muscle and adipose tissue, and plasma NEFA levels in STZ-DIO mice after treatment with vehicle, GCGRi–insulin or I501 by s.c. injection at day 21 in experiment F. (**a**) Blood glucose levels measured before treatment (i.e. *t*=0 min), at 3 h and at 3 h 45 min after treatment on day 21. Values are means ± SD (*n*=22 for all treatments). The blood glucose level in mice with diet-induced obesity is generally between 6 and 9 mmol/l, as indicated by the horizontal lines. The STZ-DIO mice were exposed to 2-DG from 3 h after treatment until euthanasia at 3 h 45 min, as indicated by the grey area. (**b**) 2-DG content in skeletal muscle samples. Symbols indicate observations from individual animals, horizontal lines indicate means, and error bars indicate SD. **p<0.01 vs the vehicle-treated group, ***p<0.001 vs the vehicle-treated group. ^†^*p*<0.05 vs the I501-treated group. (**c**) 2-DG content in adipose tissue. Symbols indicate observations from individual animals, horizontal lines indicate means, and error bars indicate SD. ^†^*p*<0.05 vs the I501-treated group. (**d**) Plasma NEFA levels in samples collected immediately before euthanasia (at 3 h 45 min after the last treatment). Symbols indicate observations from individual animals, horizontal lines indicate means, and error bars indicate SD. **p<0.01 vs the vehicle-treated group. ^†^*p*<0.05 vs the I501-treated group

At matched effects on the whole-body blood glucose level (Fig. 4a), GCGRi–insulin stimulated significantly less uptake of 2-deoxy-glucose in skeletal muscle (*p*=0.0128; Fig. 4b) and adipose tissue (*p*=0.0378; Fig. 4c) than the basal insulin comparator I501. Furthermore, GCGRi–insulin decreased plasma levels of NEFA significantly less than I501 did (*p*=0.0475; Fig. 4d). Taken together, these data demonstrated that GCGRi–insulin had a diminished effect in skeletal muscle and adipose tissue, which suggests a liver-preferential effect of GCGRi–insulin in vivo.

### Compared with conventional insulin, subchronic treatment with GCGRi–insulin resulted in elevation of liver TAGs and plasma ALT activity, and caused alpha cell hyperplasia

**Fig. 5:**
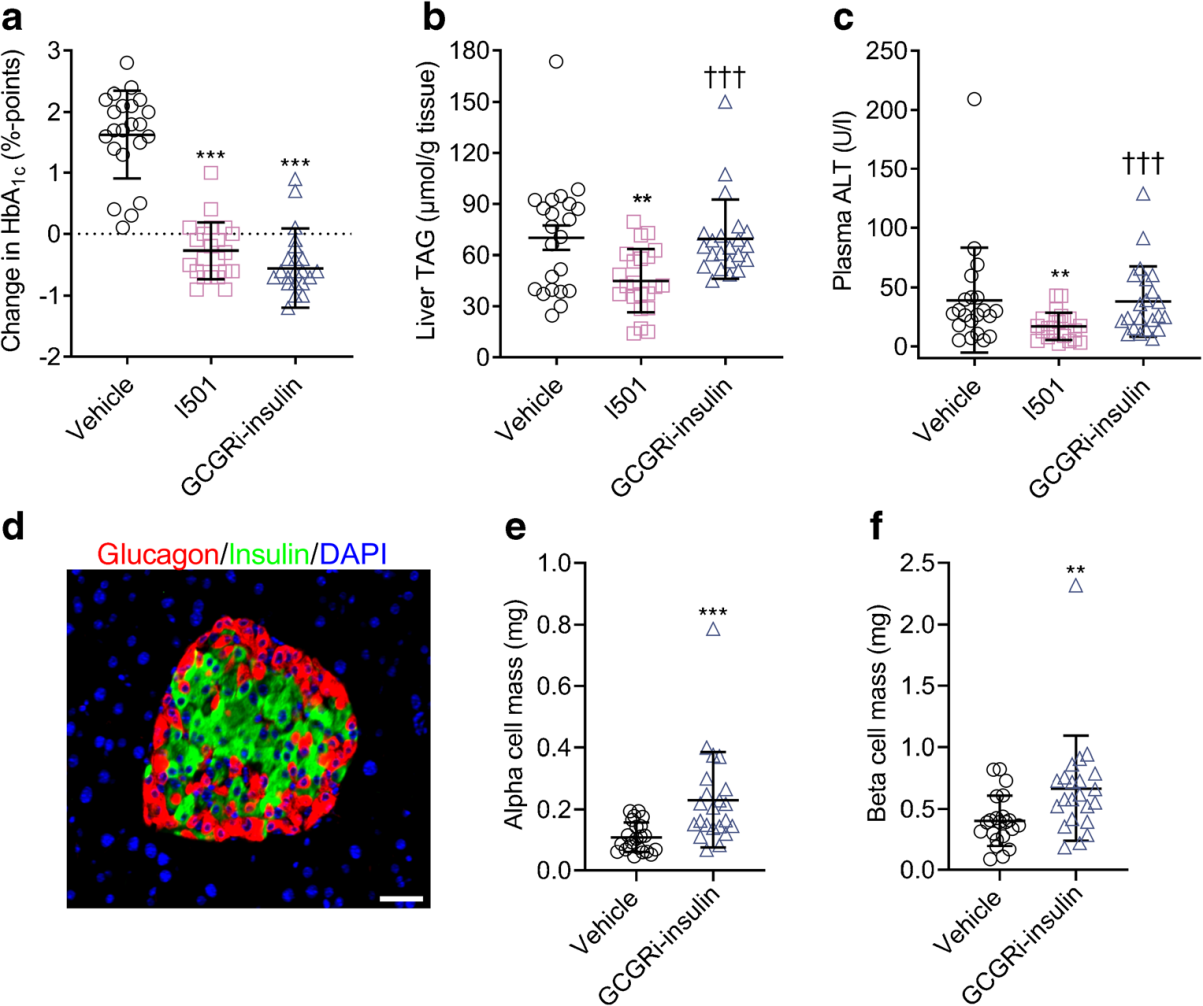
Effects of subchronic treatment with vehicle (*n*=22), GCGRi–insulin (*n*=22) or conventional I501 (*n*=22), administered by s.c. injection, for 3 weeks in STZ-DIO mice (experiment F). (**a**) Change in HbA_1c_ (%) from day 1 to day 21. Symbols indicate observations from individual animals, horizontal lines indicate means, and error bars indicate SD. ***p<0.001 vs the vehicle-treated group. (**b**) Liver TAG levels at the end of the study. Symbols indicate observations from individual animals, horizontal lines indicate means, and error bars indicate SD. **p<0.01 vs the vehicle-treated group. ^†††^*p*<0.001 vs the I501-treated group. (**c**) Plasma ALT levels at the end of the study. Symbols indicate observations from individual animals, horizontal lines indicate means, and error bars indicate SD. **p<0.01 vs the vehicle-treated group. ^†††^*p*<0.001 vs the I501-treated group. (**d**) Representative picture of an islet of Langerhans in a pancreas from an STZ-DIO mouse. Alpha cells were identified by positive staining for glucagon (red), beta cells were identified by positive staining for insulin (green), and nuclei were identified by DAPI staining (blue). White scale bar, 50 μm. (**e**) Pancreatic alpha cell mass quantified at the end of the study. Symbols indicate observations from individual animals, horizontal lines indicate means, and error bars indicate SD. ***p<0.001 vs the vehicle-treated group. (**f**) Pancreatic beta cell mass quantified at the end of the study. Symbols indicate observations from individual animals, horizontal lines indicate means, and error bars indicate SD. **p<0.01 vs the vehicle-treated group

Treatment of STZ-DIO mice for 3 weeks with GCGRi–insulin or I501 resulted in comparable reductions of HbA_1c_ in STZ-DIO mice compared with vehicle (Fig. 5a). As GCGRi–insulin and I501 had matched effects on blood glucose, it is reasonable to compare the GCGRi–insulin-treated group directly with the I501-treated group for other key variables that are known to be influenced by the level of glycaemic control in the mice. As expected for a conventional insulin treatment, I501 decreased liver TAG compared with vehicle (*p*=0.0033; Fig. 5b). However, in the group treated with GCGRi–insulin, liver TAG levels were significantly increased by approximately 60% compared with I501 (*p*=0.0008), but were not significantly different from those observed in mice treated with vehicle. I501 reduced plasma ALT compared with the vehicle-treated mice (*p*=0.0012; Fig. 5c), whereas plasma ALT activity in GCGRi–insulin-treated mice was significantly increased by approximately twofold compared with the I501-treated group (*p*=0.0002), but was not significantly different from that in the vehicle-treated group. Finally, treatment with GCGRi–insulin resulted in approximately twofold increased amounts of alpha cells (*p*<0.0001; Fig. 5e) and approximately 1.7-fold increased amounts of beta cells (*p*=0.0049; Fig. 5f) compared with the vehicle-treated group. In other similar experiments, I501 did not influence alpha or beta cell mass significantly (data not shown).

## Discussion

It is estimated that only approximately 64% of individuals with diabetes achieve individualised HbA_1c_ targets [36], and a recent study reported that approximately 79% of adults with type 1 diabetes fail to obtain the recommended target HbA_1c_ of <53 mmol/mol (7%) [37]. Hypoglycaemia is the major limiting factor in glycaemic management of type 1 and type 2 diabetes [10], and increased incidence of severe hypoglycaemia is associated with a higher risk of major adverse cardiovascular events, possibly due to rhythm abnormalities [9, 38, 39]. Thus, while better glucose regulation is needed to reduce the risk of both micro- and macrovascular complications, the risk of hypoglycaemia remains a limiting factor in insulin-dependent patients. A novel treatment that reduces the incidence of hypoglycaemia would therefore be very attractive. In this study, the novel dual-acting liver-preferential GCGRi–insulin molecule combined high potency under hyper- and normoglycaemic conditions with a dramatically reduced potency under hypoglycaemic conditions. Such a molecule would probably enable improved glycaemic control in individuals with diabetes, without increased risk of hypoglycaemia.

Several pharmaceutical companies have pursued development of GCGR antagonists (using various molecular formats), which have been shown to improve glucose regulation without increasing the risk of hypoglycaemia in patients with type 1 diabetes as well as those with type 2 diabetes [40]. These beneficial effects were also present when a GCGR antagonist was combined with basal and prandial insulin treatment in type 1 diabetes patients [20]. The liver-preferential BIL also reduced hypoglycaemia in type 2 diabetes patients [22]. Combination of glucagon antagonism and liver-preferential insulin action in a dual-acting molecule has not been explored previously in clinical trials. We explored the effects of such a molecule in preclinical models that have high translational value with respect to effects on blood glucose regulation, and the results indicate that this novel treatment concept would have considerable benefits for individuals with diabetes.

However, the GCGRi–insulin molecule also displayed potential safety concerns in animal models. Treatment of STZ-DIO mice with GCGRi–insulin for 3 weeks did not reduce liver TAG levels and plasma ALT activity, in contrast to conventional insulin treatment. The relative increase in liver TAG levels compared to mice treated with I501 is in good agreement with previous findings in rodents and individuals with type 2 diabetes [21, 41], and may be caused by inhibition of GCGR signalling, as it is known that glucagon increases hepatic lipolysis and oxidation of fatty acids [42, 43]. However, it is also possible that the increase in liver TAG is a result of the liver-preferential insulin effect of the GCGRi–insulin molecule. The liver-preferential BIL failed to decrease liver TAG levels in insulin-naive patients, and increased liver TAG levels when BIL replaced conventional insulin treatment [23], probably because of reduced inhibition of lipolysis in the adipose tissue [34].

Inhibition of GCGR in type 2 diabetes patients also increased plasma ALT activity [18, 19, 21]. This elevation was reversible, as ALT activity was normalised after termination of treatment. Elevation of plasma ALT has been suggested to be an effect of the increased amount of fat in the liver [21]. However, glucagon is also known to have an important role in regulation of amino acid metabolism [44]. GCGR knockout mice and humans with a mutation resulting in an inactive GCGR had hyper-aminoacidaemia, with particularly high levels of alanine in plasma [45, 46]. Elevation of plasma ALT may therefore be a result of effects of the GCGRi–insulin molecule on amino acid metabolism. It is also possible that the liver-preferential insulin action may contribute to the increased ALT activity. Individuals with diabetes treated with BIL had increased plasma ALT activity compared to patients treated with conventional insulin [23], but the mechanism behind this effect is not clear.

The GCGRi component in the GCGRi–insulin molecule also induced a modest hyperplasia of alpha cells (approximately twofold) compared with vehicle-treated STZ-DIO mice. Alpha cell hyperplasia has also been observed in previous animal studies with GCGR antagonists, and there is concern that such hyperplasia is a pre-neoplastic lesion that eventually could undergo malignant transformation, as reported in GCGR knockout mice and a human patient with a loss-of-function mutation in the GCGR, in which dramatically increased glucagon levels and alpha cell hyperplasia were observed [47–49]. However, treatment of hyperglycaemic mice with a GCGR antagonist had only a mild effect on plasma glucagon levels, and alpha cell numbers per islet only increased approximately 1.3-fold [50]. This is comparable to the twofold increase in alpha cell mass observed in the present study, and also in agreement with the effect observed in a clinical trial with a GCGR antagonist, in which plasma glucagon levels did not increase above approximately 200 pmol/l [18]. Taken together, GCGRi–insulin is comparable to other GCGR antagonists in terms of its effect on alpha cell hyperplasia in animal models, and the combination with insulin did not reduce this effect. Whether the elevated levels of glucagon remain a safety concern for GCGR inhibition therapies must be addressed in longer clinical studies.

A reduced capability to rescue hypoglycaemia induced by accidental overdose with insulin is a special safety concern for combined treatment with insulin and glucagon antagonists [44]. Surprisingly, treatment with the GCGRi–insulin molecule resulted in an improved effect of treatment with exogenous glucagon as well as an improved effect of the spontaneous counter-regulatory response to hypoglycaemia, probably because the GCGRi inhibited glycogenolysis in the liver, so a larger amount of glycogen was available for glycogenolysis during hypoglycaemia. Based on the present data from an animal model, concerns relating to GCGRi regarding insulin-induced hypoglycaemia therefore appear to be less relevant.

In conclusion, combination of GCGR inhibition and liver-preferential insulin action in one molecule did result in improved blood glucose regulation, but the potential safety concerns (alpha cell hyperplasia and increased levels of liver fat and plasma ALT activity) persisted. While it is not understood how a modest and reversible increase in liver TAG and plasma ALT may influence progression of non-alcoholic fatty liver disease, these potential safety concerns cannot be ignored. In order to clear a dual-acting molecule comprising glucagon inhibition and liver-preferential insulin action of these safety concerns, the compound should resemble conventional insulin in its effect on liver TAGs and liver enzymes, and preferably have no effect on alpha cell mass.

## Supplementary information

ESM (PDF 221 kb)

## Data Availability

The datasets generated and analysed during the current study are available from the corresponding author upon reasonable request.

## Abbreviations

ALT: Alanine aminotransferase
BHK: Baby hamster kidney
BIL: Basal insulin lispro
EC_50_: Half maximal effective concentration
Fab: Fragment antigen-binding region
GCGR: Glucagon receptor
GCGRi: Glucagon receptor inhibitor
HI: Human insulin
hIR-A: Human insulin receptor isoform A
hIR-B: Human insulin receptor isoform B
I501: Insulin 501
I700: Insulin 700
IR: Insulin receptor
NC: Negative control
STZ: Streptozotocin
STZ-DIO: Streptozotocin-induced hyperglycaemia and diet-induced obesity
TAG: Triacylglycerol
