# Supplementary material for: Preclinical exploration of combined glucagon inhibition and liver-preferential insulin for treatment of diabetes using in vitro assays and rat and mouse models
Source: Diabetologia. 2022 Nov 21;66(2):376–89. doi: 10.1007/s00125-022-05828-w (PMC9807490; doi:10.1007/s00125-022-05828-w)
Supplement: Supplementary file 1 — (PDF 221 kb) [file 125_2022_5828_MOESM1_ESM.pdf]

Electronic Supplementary Material (ESM) to manuscript: "Preclinical combination of glucagon inhibition and liver-preferential insulin for treatment of diabetes using in vitro assays and rat and mouse models"

ESM Methods

Compounds

The dual-acting GCGRi-insulin fusion protein used in this study was composed of a human inhibitory anti-GCGR Fab fragment including heavy chain (HC) and light chain (LC) variable regions from the anti-GCGR antibody called A-3 in US patent US-7947809-B2 [1], connected to a human insulin variant by a 200 amino acid linker (Figure 1). The variable HC was fused to the constant region of human immunoglobulin gamma 4 (IgG4 CH1; UniProt P01861; 1-101) and harbours a C-terminal HPC4 (EDQVDPRLIDGK) purification tag. The variable LC was fused to the human immunoglobulin light chain kappa constant region (IGKC) (UniProt P01834) and connected by a 50xGQAP linker to residue B1 of a single chain insulin variant precursor (consisting of B-chain (B1-B29) linked to A-chain (A1-A21) by a connecting peptide TGLGSGK). The two antibody chains were held together by a disulfide bridge between the IgG4 CH1 and IGKC. The Fab fragment was selected based on its high affinity and high inhibitor potency to ensure homing of the compound to the liver and inhibition of GCGR (Table 1). For production, the anti-GCGR-insulin fusion construct was cloned into a Chinese Hamster Ovary host cell line expression system comprising expression vectors carrying the glutamine synthetase (GS) expression cassette. Plasmid integrity was verified by sequencing. This expression system enables the development of stably transfected cell lines using selection pressure based on glutamine-free medium together with GS inhibition using L-Methionine sulfoximine. Selected producer clones were upscaled for fed-batch cultivation in bioreactor at 1 l to 50 l scale using a pre-defined proprietary cell culture medium

(Novo Nordisk). Cell viability was kept high during the first 5 to 10 days of cultivation with a progressive decrease following this until cell culture harvest at day 14. Here, cell supernatants were cleared by centrifugation and/or depth-filter filtration depending on cultivation scale before proceeding with chromatography-based protein purification.

A reference Fab-insulin fusion protein harbouring the unspecific aTNP Fab fragment instead of the anti-GCGR Fab fragment described above for the dual-acting GCGRi-insulin, was constructed to serve as a negative control (NC). This construct was generated in the mammalian cell expression vector pTT5 as described previously [2] and plasmids verified by sequencing. The aTNP Fab-insulin fusion reference protein was produced in smaller scale by transient expression in Expi293F™ cells (ThermoFisher Scientific, Denmark) according to standard protocol.

Harvest material from large-scale fed-batch cultivations and smaller-scale transient expressions was purified using Capto L affinity resin (Cytiva, GE Healthcare, Danmark) or anti-HPC4 resin. For purification by Capto L affinity, resin was washed in equilibration buffer (10 mmol/l Na-phosphate, pH 7.5, 50 mmol/l NaCl) and elution performed in 20 mmol/l formic acid pH 3.5. A purification protocol for HPC4-tagged recombinant protein was established using a calcium-dependent IgG anti-HPC4 antibody as reported previously [3]. Recombinantly produced anti-HPC4 antibody was coupled to CNBr-Sepharose FF resin according to vendors protocol (Cytiva, GE Healthcare, Danmark) and the following buffer system used for purification: equilibration buffer (20 mmol/l HEPES, 150 mmol/l NaCl, 2 mmol/l $CaCl_2$, pH 7.4), wash buffer (20 mmol/l HEPES, 1 mol/l NaCl, 2 mmol/l $CaCl_2$, pH 7.4) and elution buffer (20 mmol/l HEPES, 150 mmol/l NaCl, 20 mmol/l EDTA, pH 7.4).

The capture pool was concentrated using a 10 kD spin membrane/centrifuge or a 10 kD UF/DF membrane/Akta CrossFlow. Concentrated protein was subsequently treated with *Achromobacter lyticus* protease I, ALP (EC 3.4.21.50), specific for lysyl peptide bonds, under controlled conditions (using a 0.2-1% w/w ratio of ALP in solution in 20 mmol/l HEPES, 5 mmol/l EDTA, 100 mmol/l NaCl, pH 7.5) to convert the single chain insulin precursor into the active mature two-chain insulin form. Size exclusion chromatography using Superdex 200 resin column (Cytiva, GE Healthcare, Danmark) and buffer (25 mmol/l histidine, pH 6.5, 150 mmol/l NaCl) was performed to remove C-peptide and residual ALP. Protein integrity and purity was analyzed using a size-exclusion high-performance liquid chromatographic method on an Agilent LC 1100/1200 system with BIOSEP-SEC-53000 300×7.8 mm column (Phenomenex, Denmark). Molecular mass of purified proteins was determined by mass spectrometry on a 6280 Agilent system (Agilent Technologies, Denmark). Final protein concentration was determined using a NanoDrop™ spectrophotometer (ThermoFisher Scientific, Denmark) with theoretical calculated extinction coefficients

Insulin analogues I501, I700 and BIL used as comparators in this study were produced by Novo Nordisk. Insulin I501 is desB30 human insulin conjugated at B29Lys with octadecandioic acid via a γ-glutamic acid linker. Insulin I700 is human insulin conjugated at B29Lys with hexadecandioic acid via a γ-glutamic acid linker. Basal insulin lispro (BIL) (insulin peglispro; LY2605541) is insulin lispro (B28Lys, B29Pro insulin) PEGylated with a 20 kDa PEG moiety at the ε-amino group of B28Lys [4]. BIL is a basal insulin with a liver-preferential action [5].

Insulin receptor and glucagon receptor binding

Affinities for human insulin receptor isoform A and B (hIR-A and hIR-B) were determined by scintillation proximity radio-ligand competition binding assays as previously reported [6].

Competitive 125I-glucagon binding was performed on plasma membranes prepared from Baby hamster kidney (BHK) cells expressing the human GCGR and CRE firefly luciferase. BHK cells (mycoplasma free and purchased from ATCC, VA, USA) stably over-expressing human glucagon receptor and cAMP response element (Cre) coupled to firefly luciferase (Cre-luciferase) were generated at Novo Nordisk. The cells were grown at 37 °C and 5% (vol./vol.) CO_2 in DMEM supplemented with 100 IU/ml penicillin, 100 µL/ml streptomycin, 10% (vol./vol.) fetal calf serum and 1 mg/ml geneticin G-418. Membranes were suspended in an assay buffer consisting of 50 mmol/l HEPES (pH 7.4) containing 5 mmol/l $MgCl_2$ (Sigma), 1 mmol/l $CaCl_2$ (Sigma), 0.02% (vol./vol.) Tween-20 (Sigma) and 0.1 % (wt./vol.) Ovalbumin (Sigma) to a concentration of 0.06 mg/ml and kept on ice until used. Wheat germ agglutinin SPA beads (Perkin Elmer) were suspended in the same buffer at 5 mg/ml just prior to addition. Compounds to be tested were diluted in the assay buffer and 25 µl aliquot of the compound dilutions were added to the OptiplateTM-96 plate (Perkin Elmer) to reach final assay concentrations of 0-1 µmol/l together with 50 µl assay buffer, 50 µl of the cell membrane preparation, 50 µl of the SPA bead suspension and 25 µl of ^{125}I-glucagon 480 pmol/l (final concentration 60 pmol/l, 37MBq/l, Novo Nordisk). The plates were then incubated for 2 h at 30 °C. The assay was terminated by centrifugation of the assay plate for 10 min at 1500 rpm. The plate was read in a TopCount NXT instrument (Perkin-Elmer). Data were imported into GraphPad Prism and IC50 values were determined using the following nonlinear regression equation: log (inhibitor) vs. response (four parameters).

Inhibition of glucagon receptor signalling in BHK cells

A cAMP-sensitive luciferase reporter gene assay was applied to further characterize receptor functionality. Frozen aliquots of BHK cells expressing the mouse GCGR and Cre-firefly luciferase were thawed, washed twice in PBS, and suspended in the assay buffer (DMEM w/o phenol red (Gibco), 10 mmol/l HEPES (Gibco), 1× GlutaMAX (Gibco), 1% (wt./vol.) Ovalbumin (Sigma), and 0.1% (wt./vol.) Pluronic F-68 (Gibco). Cells were plated out into black 96-well plates (NUNC) at 5000 cells/well in a volume of 50 µl. Compounds to be tested were diluted in the assay buffer together with glucagon (Novo Nordisk) and a 50 µl aliquot transferred to the plate containing the cells to reach final assay concentrations of 0-10 µmol/l of the compounds and 20 pmol/l concentration of glucagon. The plate was incubated for 3h at 5% CO_2 (vol./vol.) at 37 °C, then moved to room temperature 15 min prior to adding 100 µl of Steadylite plus reagent (PerkinElmer). The plate was covered to protect it from light and shaken at room temperature for 30 min. The plate was read in a TopCount NXT instrument (Perkin-Elmer). Data were imported into GraphPad Prism and IC50 values were determined using the following nonlinear regression equation: log(inhibitor) vs response (four parameters).

Insulin receptor signalling in primary rat hepatocytes

Hepatocytes from ad libitum-fed male Sprague-Dawley rats (200 g) were isolated in situ by a two-step perfusion technique as described previously [7, 8] and incubated in assay media (M199 culture medium (5.5 mmol/l glucose, 100 units/ml penicillin and 100 mg/ml streptomycin and 4 mg/ml decadron supplemented with 0.1% (vol./vol.) fetal calf serum (FCS) and 1 nmol/l human insulin (HI). The next day media was changed to assay media supplemented with 0.1% (vol./vol.) human serum albumin (HSA) and increasing concentrations of either HI or dual GCGRi-insulin compound

for 15 min at 37 °C. Cells were then lysed in 100 µl lysis buffer (Cell Extraction Buffer, Invitrogen FNN0011) supplemented with protease inhibitor cocktail (Sigma) and protease inhibitor AEBSF (Sigma). Subsequently, phosphorylation of insulin receptor (p-Tyr1150/1151) and Akt (p-Ser473) were quantified using the immunoassay AlphaScreen SureFire Assay Kit (Perkin Elmer, cat #TGRIRS500, TGRA4S10k) according to manufacture instructions. Alpha counts obtained from Alpha Technology-compatible plate reader were fitted to a log-dose response profile using an unconstrained four-parameter logistic equation in Graph Pad Prism.

Inhibition of GCGR signalling in primary rat hepatocytes

Hepatocytes from ad libitum-fed male Sprague-Dawley rats (200 g) were isolated in situ by a two-step perfusion technique as described previously [7, 8]. Cell viability, determined with a NucleoCounter (Chemometec), was consistently greater than 85%. Hepatocytes were diluted to a cell density of 0.5× 10^6 cells/ml in basal medium (Medium 199 with glutamax and 5.5 mmol/l glucose (Gibco) supplemented with 100 nmol/l dexamethasone (Sigma), 1% (wt./vol.) penicillin streptomycin (Gibco), 1 nmol/l HI (Novo Nordisk) and 4% (vol./vol.) FBS (Gibco)) and plated in collagen-coated plates (Corning BioCoat) for glycogenesis assay (50.000 cells/well). To remove dead cells, the medium was changed to basal medium with 0.1% (vol./vol.) FBS 3 h after initial plating. The following day the medium was removed and replaced with basal medium in the presence of 0.1% (vol./vol.) HSA (Sigma) and 14.5 mmol/l glucose (final concentration 20 mmol/l) and incubated for 24 h with compounds in concentrations from 0-1 µmol/l. Cells were then washed with PBS and flash frozen with liquid nitrogen.

For cAMP measurement, hepatocytes were resuspended in Stimulation Buffer with IBMX from the Flashplate kit (Perkin-Elmer) to 1 mio cells/ml, seeded in a Flashplate (50 µl/well) and

incubated with compounds for 30 min at RT. The compounds were diluted in assay buffer (HBSS w. Ca^{2+} and Mg^{2+} (Gibco) with 10 mmol/l Hepes (Gibco), 0.1% (wt./vol.) Pluronic-F68 (Gibco)) with 2% (vol./vol.) HSA (Sigma) to a concentration of 4X the final, and 25 µl was added to each well in the Flashplate. The cAMP standard curve was diluted in Stimulation buffer and added to wells with 50 µl assay buffer. After the 30 min incubation, 25 µl glucagon (Novo Nordisk) was added to a final concentration of 3 nmol/l. The plate was then incubated at 37 °C, 5% (vol./vol.) CO_2 for 30 mins. The cells were lysed by adding 100 µl Detection buffer with ^{125}I-cAMP tracer from the kit. The plate was sealed and stored at RT in the dark for 2 h. Then the plate was counted using Topcounter NXT (Perkin-Elmer). Data were imported into GraphPad Prism and EC50 values were determined using the following nonlinear regression equation: log(inhibitor) vs response (four parameters).

Glycogen synthesis in primary hepatocytes

Primary rat hepatocytes were incubated in medium with 14.5 mmol/l glucose for 24 h with increasing concentrations of compounds. Cells were then lysed by freezing in liquid nitrogen. The plates with lysed cells were subsequently thawed and incubated with 70 µl/well amyloglucosidase (to convert glycogen to glucose) diluted 100X in 0.2 mol/l Na-acetate (Sigma) pH 4.8 for 2.5 h at 40 °C with shaking. 40 µl were transferred to a clear 96 well plate (Nunc Immunoplate) and pH neutralized with 10 µl 0.25 mol/l NaOH (Sigma). Glucose was measured with a kit (BioVision). A glucose standard curve was included on each plate using 50 µl per well. Assay mix from the kit (50 µl) was added to each well and the plates were incubated at 37°C for 10-15 min. Then the absorbance at 570 nm was measured. The cellular glucose (i.e., originating from glycogen) content was calculated with linear regression using the glucose standard curve. Data were imported into

GraphPad Prism and EC50 values were determined using the following nonlinear regression equation: log(agonist) vs response (four parameters).

Lipogenesis in primary mouse adipocytes

In primary mouse adipocytes isolated from epididymal fat pads, the effect of GCGRi-insulin and NC on lipogenesis was determined by measuring the incorporation of [3H]-labelled glucose into fat as described previously [9].

Animal experiments

Details regarding designs of the animals experiments (A-F) included in this study are shown in ESM Table 1, including descriptions of group size determinations/calculations, randomization and blinding. All animals were acclimatized for minimum a week prior to initiation of experimental procedures, and all animals were housed under specific pathogen free conditions in type IV Makrolon® cages with floor area 1800 cm^2. Mice were housed ten animals per cage. Rats were housed five animals per cage. After surgical cannulation of rats (experiment C, D E) the animals were single-housed. All animals were treatment naïve when the experiments were started.

For acute treatment with GCGRi-insulin, NC or GCGRi, male Sprague Dawley rats (NTac:SD) (≈300 g upon arrival from Taconic) (experiment A) were made hyperglycaemic by treatment with 65 mg/kg streptozotocin (STZ). Two weeks later these hyperglycaemic streptozotocin-induced rats (STZ-rats) were treated with vehicle, GCGRi-insulin, NC or GCGRi by i.v. injection. Blood glucose was measured right before treatment and blood samples were collected from the tongue vein at 30 min, 1, 2, 4, 6 and 8 h after treatment for assessment of blood glucose

and plasma concentrations of test compounds. Blood glucose and plasma exposure of test compounds were assessed as described previously [10].

Male Db/db mice (BKS.Cg-m+/+*Lepr*^{db}/BomTac) for acute treatment with GCGRi-insulin or I700 (experiment B) were purchased from Taconic at age of nine weeks. Mice were treated with GCGRi-insulin or I700 by i.v. injection and blood glucose was measured right before treatment and 30, 60, 90, 120, 150, 210 and 240 min after treatment. Blood glucose was measured as described for the rat study.

Male Sprague Dawley rats (Crl:CD(SD)) (≈350 g upon arrival from Charles River, Germany) were used in experiments where GCGRi-insulin and reference compounds were administered by i.v. infusion (experiment C, D and E). The rats were cannulated and the blood sampling and infusion techniques were conducted as previously described [11]. The experiments consisted of a 30 min basal period and a 90 min period of titrated intravenous infusions of HI, I700, BIL or GCGRi-insulin. Arterial plasma glucose concentrations were measured at 10 min intervals throughout while variable molar rates (pmol/kg/min) of insulin or GCGRi-insulin were adjusted accordantly to reach glycaemic targets of 3 or 4 mmol/l. After reaching the glycaemic targets, the insulin and GCGRi-insulin infusions were kept constant at the individual rate, and an i.v. bolus of HI (1.1 nmol/kg), glucagon (10 nmol/kg) or vehicle was given at 90 min and the plasma glucose concentrations were monitored for the following 90 min, where the experiment was terminated and the rats euthanised. The constant molar infusion rates of HI, I700, BIL or GCGRi-insulin from 90-180 min in rats given vehicle at 90 min in the 3 and 4 mmol/l glucose studies were used for a statistical post-hoc analysis (Table 2).

The effects of GCGRi-insulin and I501 on glucose uptake in adipose tissue and skeletal muscle, HbA_{1c}, plasma FFA, plasma ALT, liver TAG levels and pancreatic alpha cell mass

(experiment F) were explored in male C57Bl/6 mice (000664 C57BL/6J) with diet-induced obesity (DIO) purchased from Jackson (ME, USA), which at age of 14 weeks were made hyperglycaemic with STZ (Sigma) 150 mg/kg (STZ-DIO mice). Two weeks after treatment with STZ, treatment with vehicle, GCGRi-insulin or I501 twice daily by s.c. injection was initiated and continued for three weeks. At the last day of treatment, blood glucose was measured right before treatment and after 3 h. Immediately thereafter, ^{14}C-2-deoxy-glucose (2-DG, NEC495A001MC, PerkinElmer, Denmark), 4 ml/kg, (equivalent of 0.01 µCi/g bodyweight) was administered by s.c. injection in the right flank. Forty-five min later a larger blood sample was collected for assessment of blood glucose and plasma FFA, the animals were euthanised and samples from the s.c. fat (lower flank), the gastrocnemius muscle and liver were collected and snap-frozen in liquid nitrogen. Furthermore, the pancreas was collected and fixed in 10% (wt./vol.) neutral buffered formalin (VWR, Denmark). Blood glucose was measured as described above. Plasma FFA levels, plasma ALT levels and liver TAG levels were assessed as described previously [12]. 2-DG content in muscle and fat was quantified as described previously [13].

Histology

Pancreas was collected, weighed and fixed in 10% (wt./vol.) natural buffered formalin. The tissue was processed to paraffin as previously described by Paulsen et al. [14], however pancreas was cut into four slabs for stereological assessment of alpha and beta cell mass and embedded in paraffin. Sections of 3 µm thickness were cut and stained with guinea pig anti-insulin (1:75, A0564, Dako, Denmark) and mouse anti-glucagon (1:7800, Glu-001 (RRID: AB_2910264), Novo Nordisk, Denmark). Insulin was visualised with goat-anti-guinea pig (1:100, A11073, Invitrogen, Denmark) followed by donkey-anti-goat-Alexa 488 (1:100, 705-545-147, Jackson ImmunoResearch, PA,

USA). Glucagon was visualised with donkey-anti-mouse-Cy5 (1:100, 715-175-151, Jackson ImmunoResearch). The slides were counterstained with DAPI (1:1000, H3569, Invitrogen) and scanned at 10x magnification on a VS120 slide scanner (Olympus, Germany). An image analysis algorithm was developed in VIS (Visiopharm, Denmark) to detect total tissue area, glucagon area (alpha cells) and insulin area (beta cells). Alpha cell mass was calculated as:

$$alpha\ cell\ mass\ (mg) = \frac{glucagon\ area\ (\mu m^2)}{total\ tissue\ area\ \ (\mu m^2)} * pancreas\ weight\ (mg)$$

Beta cell mass was calculated as:

$$beta\ cell\ mass\ (mg) = \frac{insulin\ area\ (\mu m^2)}{total\ tissue\ area\ \ (\mu m^2)} * pancreas\ weight\ (mg)$$

Statistical analysis

Statistical analysis was performed using GraphPad Prism (GraphPad Software, CA, USA), JMP (SAS Institute, NC, USA) or SAS software (SAS Institute). All in vitro data were fitted to a four-parameter logistic model, and the estimated EC_{50}/IC_{50} values were subsequently used for calculation of geometric mean values and 95% CI. In vivo data were analysed using general linear models, followed by pairwise comparison of treatment groups, with Tukey's adjustment for multiple parallel pairwise comparisons. Data were log-transformed prior to analysis if assumptions of normal distribution and/or variance homogeneity were not fulfilled. The individual molar infusion rates required to obtain plasma glucose of 3 or 4 mmol/l in experiments C, D and E were used in a post hoc analysis. All data were log-transformed and analysed using two-way ANOVA with interaction between the factors compound and plasma glucose target level. Separate variances were estimated to take variance inhomogeneity into account. Furthermore, differences between each compound and HI were estimated on the log scale. The double difference (i.e. [compound X vs HI

at 3 mmol/l] vs [compound X vs HI at 4 mmol/l]) was also estimated on the log scale. Estimated differences and double differences on the log scale were back-transformed to ratios (i.e. [compound X at 3 mmol/l/HI at 3 mmol/l]/[compound X at 4 mmol/l/HI at 4 mmol/l]). In all analyses, p values < 0.05 were considered statistically significant.

ESM Table 1: Details of animal experiments

Study	Aim	Design of experiment
A	Compare effect of GCGRi-insulin, NC and GCGRi on blood glucose in STZ-rats.	Male Sprague-Dawley rats (≈300 g upon arrival from Taconic, Denmark) were made hyperglycaemic with streptozotocin 65 mg/kg. Two weeks later, rats were treated with either vehicle (n=5), GCGRi-insulin 10 nmol/kg (n=5), GCGRi-insulin 30 nmol/kg (n=5), NC 10 nmol/kg (n=5) or NC 30 nmol/kg (n=3) by i.v. injection. Blood glucose was measured right before treatment, and blood samples for assessment of blood glucose and plasma exposure of test compounds were collected 30 min, 1, 2, 4, 6 and 8 h after treatment. On a separate experimental day other hyperglycaemic rats were treated with either vehicle (n=5) or GCGRi 60 nmol/kg (n=5) by i.v. injection. Blood glucose was measured right before treatment, and blood samples for assessment of blood glucose and plasma exposure of test compounds were collected 30 min, 1, 2, 3, 4 and 8 h after treatment.<br>Five animals were included per group, because previous sample size calculations had revealed this allowed for a minimal detectable difference in blood glucose between two groups of ≈5-6 mmol/l, which was judged as sufficiently accurate for the purpose of this experiment. Animals were randomized to treatment groups based on the blood glucose measured in the morning the day before the experiment. Based on this blood glucose measurement, the animals were sorted in ascending order. The first animal was then allocated to group 1, the second animal to group 2, etc. Thereby the average blood glucose was also comparable between the different groups.<br>Type of treatment was not blinded for the operators treating the rats and collecting blood samples, because it was evaluated that it was not possible for the operators to influence the endpoints in the experiment (plasma concentration of test compounds and/or blood glucose). No animals were excluded from the experiment or data analysis.
B	Compare effect of GCGRi-insulin and I700 on blood glucose in db/db mice.	Male db/db mice (9 weeks old upon arrival from Taconic, Denmark) were treated with either vehicle (n=8), GCGRi-insulin 5 nmol/kg (n=5), GCGRi-insulin 20 nmol/kg (n=5), GCGRi-insulin 80 nmol/kg (n=5), GCGRi-insulin 320 nmol/kg (n=5), I700 1 nmol/kg (n=8), I700 4 nmol/kg (n=8), I700 16 nmol/kg (n=8), I700 64 nmol/kg (n=8) or I700 256 nmol/kg (n=10), administered by i.v. injection. Blood glucose was measured immediately before treatment and 30, 60, 90, 120, 150, 180 and 240 min after treatment.<br>Five to ten animals were included per group because sample size calculations showed this would allow for a minimal detectable difference in blood glucose between two groups of ≈7-4.5 mmol/l, which for the purpose of the experiment was judged as sufficiently sensitive.

		Animals were randomized to treatment groups based on their bodyweight and their blood glucose measured one or two days before they were treated with test compound, using the minimisation method described previously [15].<br>Type of treatment was not blinded for the operators treating the mice and collecting blood samples, because it was evaluated that it was not possible for the operators to influence the blood glucose measured in the blood samples. No animals were excluded from the experiment or data analysis.
C	Compare the response to glucagon between rats where hypoglycaemia was induced by constant infusion with either HI or GCGRi-insulin	Male Sprague Dawley rats (≈350 g upon arrival from Charles River, Germany) were cannulated as previously described [11]. On the day of the experiment, the rats were first given a 30 min basal period, followed by 90 min with i.v. infusion of either HI or GCGRi-insulin, in doses which were titrated in each individual animal to reach a plasma glucose of 3 mmol/l. Arterial plasma glucose concentrations were measured at 10 min intervals throughout while variable molar rates (pmol/kg/min) of insulin or GCGRi-insulin were adjusted accordantly to reach a plasma glucose of 3 mmol/l. After reaching the glycaemic target, infusion of HI or GCGRi-insulin was kept constant at the individual rate, and a bolus of glucagon (10 nmol/kg) or vehicle was then administered i.v. (HI+vehicle bolus: n=6 , HI+glucagon bolus: n=6 , GCGRi-insulin+vehicle bolus: n=6 , GCGRi-insulin + glucagon bolus: n=6). Blood glucose was then monitored every 10th min for another 90 min. Thereafter the experiment was terminated and the rats were euthanised.<br>Six animals were included per group because previous sample size calculations had revealed this allowed for a minimal detectable difference between two groups in blood glucose upon challenge with glucagon of ≈0.9 mmol/l. This was judged as sufficiently accurate for the purpose of this experiment. Animals were randomized to treatment groups by randomly picking one rat for each type of treatment on each experimental day (first randomly selected rat for group 1, second randomly selected rat for group 2, etc.). Type of treatment was not blinded for the operators treating the rats and collecting blood samples, because it was evaluated that it was not possible for the operators to influence plasma concentrations of test compounds and the plasma glucose measured in the blood samples. No animals were excluded from the experiment or data analysis.
D	Compare the response to a bolus of fast-acting insulin between rats where plasma glucose was decreased to 4 mmol/l by constant	Male Sprague Dawley rats (≈350 g upon arrival from Charles River, Germany) were cannulated as previously described [11]. On the day of the experiment, the rats were first given a 30 min basal period, followed by 90 min with i.v. infusion of either HI or GCGRi-insulin, in doses which were titrated in each individual animal to reach a plasma glucose of 4 mmol/l. Arterial plasma glucose concentrations were measured at 10 min intervals throughout this period, while variable molar rates (pmol/kg/min) of insulin or GCGRi-insulin were adjusted accordantly to reach a plasma glucose of 4 mmol/l. After reaching the glycaemic target, infusion of HI or GCGRi-insulin was kept constant at the individual rate, and a bolus of HI (1.1 nmol/kg) or vehicle was then administered i.v. (HI+vehicle bolus: n=8 ,

	infusion of either HI or GCGRi-insulin.	HI+insulin bolus: n=7 , GCGRi-insulin+vehicle bolus: n=7 , GCGRi-insulin+insulin bolus: n=8). Blood glucose was then monitored every 10th min for another 90 min. Thereafter the experiment was terminated and the rats were euthanised.<br>Seven or eight animals were included per group because previous sample size calculations had revealed this allowed for a minimal detectable difference between two groups in blood glucose upon administration of a bolus of fast-acting insulin of ≈0.5 mmol/l. which was judged as sufficiently accurate for the purpose of this experiment. Animals were randomized to treatment groups as described above for experiment C. Type of treatment was not blinded for the operators treating the rats and collecting blood samples, because it was evaluated that it was not possible for the operators to influence plasma concentrations of test compounds and the plasma glucose measured in the blood samples. No animals were excluded from the experiment.
E	Compare the response to a bolus of fast-acting insulin between rats where plasma glucose was decreased to 3 or 4 mmol/l by constant infusion of either HI, I700 or BIL.	Male Sprague Dawley rats (≈350 g upon arrival from Charles River, Germany) were cannulated as previously described [11]. On the day of the experiment, the rats were first given a 30 min basal period, followed by 90 min with i.v. infusion of either HI, I700 or BIL, in doses which were titrated in each individual animal to reach a plasma glucose of 3 mmol/l or 4 mmol/l (HI 3 mmol/l: n=8 , HI 4 mmol/l: n=6 , I700 3 mmol/l: n=9 , I700 4 mmol/l: n=6 , BIL 3 mmol/l: n=4 , BIL 4 mmol/l: n=6). Arterial plasma glucose concentrations were measured at 10 min intervals throughout this period, while variable molar rates (pmol/kg/min) of insulin or GCGRi-insulin were adjusted accordantly to reach a plasma glucose of 3 mmol/l. After reaching the glycaemic target, infusion of HI or GCGRi-insulin was kept constant at the individual rate for another 90 min during which plasma glucose was also measured every 10th minute. Thereafter the experiment was terminated and the rats were euthanised.<br>The different treatment groups included between four and nine rats because previous sample size calculations revealed this allowed for determination of a significant difference in compound infusion rates between the group treated with compound X to obtain plasma glucose of 3 mmol/l and the group treated with compound X to obtain plasma glucose of 4 mmol/l of minimum ≈2-fold. Considering that this difference for GCGRi-insulin was >4.5-fold, this was considered sufficiently accurate for the purpose of the experiment. Animals were randomized to treatment groups as described above for experiment C. Type of treatment was not blinded for the operators treating the rats and collecting blood samples, because it was evaluated that it was not possible for the operators to influence plasma concentrations of test compounds and the plasma glucose measured in the blood samples. No animals were excluded from the experiment or data analysis.
F	Compare effect of GCGRi-insulin and I501 on glucose	Male C67Bl6/J mice (12 weeks old, with 6 weeks on 60 kcal% high-fat diet, upon arrival from Jackson, US) were made hyperglycaemic with streptozotocin 150 mg/kg, after in total 8 weeks on 60 kcal% high-fat diet (Diet D12492, Research diets). Two weeks later, treatment with either vehicle

	uptake in adipose tissue and skeletal muscle, plasma NEFA, liver TAG and alpha cell mass at matched blood glucose lowering in STZ-DIO mice.	(n=22), GCGRi-insulin 60 nmol/kg (n=22) or I501 30 nmol/kg (n=22) twice daily by s.c. injection was initiated. The mice were treated for 20 days. At the 21st day, blood glucose was measured in the morning immediately before treatment with vehicle/GCGRi-inuslin/I501. Blood glucose was measured again 3 h after treatment, and all mice were then dosed with ^{14}C-2-deoxy-glucose (2-DG), 4 ml/kg (equivalent of 0.01 µCi/g bodyweight). 2-DG was administered by s.c. injection in the right flank. Forty-five min after this, a larger blood sample was collected from the tongue vein for assessment of blood glucose and plasma NEFA, and immediately after this the animals were euthanised and samples were collected from the s.c. fat in the lower flank area, the gastrocnemius muscle and the liver. Tissue samples were snap-frozen in liquid nitrogen and later used for quantification of 2DG content. Finally, the entire pancreas was collected and fixed in neutral buffered formalin.<br><br>Liver TAG content was the most important endpoint in this experiment, and therefore 22 animals were included in each group, because sample size calculations showed this allowed for a minimum detectable difference in liver TAG content between two groups of approximately 30%.<br><br>Animals were allocated to treatment groups based on their blood glucose, HbA_{1c}, fat mass and bodyweight measured the day before treatment was initiated, using the minimisation method described previously [15]. Type of treatment was not blinded for the operators treating the animals, because it was evaluated that it was not possible for the operators to influence the endpoints measured during the study. No animals were excluded from the experiment or data analysis.<br><br>For practical reasons, the experiment was performed in four blocks staggered in time. All treatment groups were represented in each block (i.e., 5 or 6 animals per treatment in each block). In the statistical analysis, the potential effect of block was taken into account by analysing each endpoint in a general linear model with the factors treatment (levels: vehicle, I501, GCGRi-insulin) and block (levels: A, B, C, D) and the possible interaction between treatment and block. No significant interaction between treatment and block was seen for any of the endpoints. For each endpoint, this model was used to compare the treatment groups to each other, in three parallel t-tests, with Tukeys adjustment for multiple parallel comparisons.

ESM References

[1] Yan H, Hu S-FS, Boone T C, Lindberg RA, inventors; Amgen Inc, assignee. Compositions and methods relating to glucagon receptor antibodies. US patent US-7947809-B2, 24th May 2011. Available from https://testpubchem.ncbi.nlm.nih.gov/patent/US-7947809-B2. Accessed 21st August 2021.

[2] Durocher Y, Perret S, Kamen A (2002) High-level and high-throughput recombinant protein production by transient transfection of suspension-growing human 293-EBNA1 cells. Nucleic Acids Res 30(2): E9. 10.1093/nar/30.2.e9

[3] Rezaie AR, Fiore MM, Neuenschwander PF, Esmon CT, Morrissey JH (1992) Expression and purification of a soluble tissue factor fusion protein with an epitope for an unusual calcium-dependent antibody. Protein Expr Purif 3(6): 453-460. 10.1016/1046-5928(92)90062-2

[4] Caparrotta TM, Evans M (2014) PEGylated insulin Lispro, (LY2605541)--a new basal insulin analogue. Diabetes Obes Metab 16(5): 388-395. 10.1111/dom.12196

[5] Moore MC, Smith MS, Sinha VP, et al. (2014) Novel PEGylated basal insulin LY2605541 has a preferential hepatic effect on glucose metabolism. Diabetes 63(2): 494-504. 10.2337/db13-0826

[6] Vienberg SG, Bouman SD, Sorensen H, et al. (2011) Receptor-isoform-selective insulin analogues give tissue-preferential effects. Biochem J 440(3): 301-308. 10.1042/BJ20110880

[7] Andersen B, Rassov A, Westergaard N, Lundgren K (1999) Inhibition of glycogenolysis in primary rat hepatocytes by 1, 4-dideoxy-1,4-imino-D-arabinitol. Biochem J 342 Pt 3: 545-550

[8] Seglen PO (1976) Preparation of isolated rat liver cells. Methods Cell Biol 13: 29-83. 10.1016/s0091-679x(08)61797-5

[9] Moody AJ, Stan MA, Stan M, Gliemann J (1974) A simple free fat cell bioassay for insulin. Horm Metab Res 6(1): 12-16. 10.1055/s-0028-1093895

[10] Hvid H, Kildegaard J, Kristensen K, et al. (2020) Intraintestinal and Parenteral Administration of an Insulin Analogue Leads to Comparable Activation of Signaling Downstream of the Insulin Receptor in the Small Intestine. J Diabetes Sci Technol 14(1): 112-119. 10.1177/1932296819855075

[11] Sorensen AR, Stidsen CE, Ribel U, et al. (2010) Insulin detemir is a fully efficacious, low affinity agonist at the insulin receptor. Diabetes Obes Metab 12(8): 665-673. 10.1111/j.1463-1326.2010.01206.x

[12] Jensen VS, Fledelius C, Zachodnik C, et al. (2021) Insulin treatment improves liver histopathology and decreases expression of inflammatory and fibrogenic genes in a hyperglycemic, dyslipidemic hamster model of NAFLD. J Transl Med 19(1): 80. 10.1186/s12967-021-02729-1

[13] Brand CL, Sturis J, Gotfredsen CF, et al. (2003) Dual PPARalpha /gamma activation provides enhanced improvement of insulin sensitivity and glycemic control in ZDF rats. Am J Physiol Endocrinol Metab 284(4): E841-854. 10.1152/ajpendo.00348.2002

[14] Paulsen SJ, Vrang N, Larsen LK, Larsen PJ, Jelsing J (2010) Stereological assessment of pancreatic beta-cell mass development in male Zucker Diabetic Fatty (ZDF) rats: correlation with pancreatic beta-cell function. J Anat 217(5): 624-630. 10.1111/j.1469-7580.2010.01285.x

[15] Altman DG, Bland JM (2005) Treatment allocation by minimisation. BMJ 330(7495): 843. 10.1136/bmj.330.7495.843
